# Effectiveness of nebulized amphotericin B to eradicate *Candida *colonization from the lower respiratory tracts of ICU patients

**DOI:** 10.1186/cc11719

**Published:** 2012-11-14

**Authors:** DSY Ong, PMC Klein Klouwenberg, MJM Bonten, OL Cremer

**Affiliations:** 1 University Medical Centre Utrecht, the Netherlands

## Background

*Candida *species are opportunistic pathogens that are ordinarily found in the human gastrointestinal tract. In critically ill patients receiving mechanical ventilation, colonization of the lower respiratory tract (LRT) with *Candida *may occur in 25 to 55% of patients. Nebulized amphotericin B (NAB) is commonly used to eradicate *Candida *from the LRT, for example as part of selective decontamination of the digestive tract protocols. However, the clinical effectiveness of this approach is unknown. Our aim was to determine the time to eradication of *Candida *from the LRT in mechanically ventilated ICU patients receiving and not receiving inhalation therapy with NAB.

## Methods

We included patients admitted to the ICU of the University Medical Center Utrecht from November 2007 until February 2012. We excluded patients with a length of stay <72 hours and patients receiving systemic antifungal treatment. Microbiological screening for *Candida *colonization was performed on admission and twice weekly, and samples were processed according to a standardized protocol. Samples obtained in the first 72 hours of ICU admission were discarded since positive samples obtained on admission were not an indication to start amphotericin B. Colonization was defined as the presence of *Candida *in two or more consecutive samples obtained on different days. Decolonization was defined as the absence of *Candida *in two consecutive samples, or as the absence of Candida in the last available sample before extubation or discharge. Only the first episode of *Candida *colonization per admission was used for analysis. Hazard ratios of eradication of *Candida *colonization in treated patients compared with nontreated patients were determined using Cox regression analysis.

## Results

**Figure 1:**
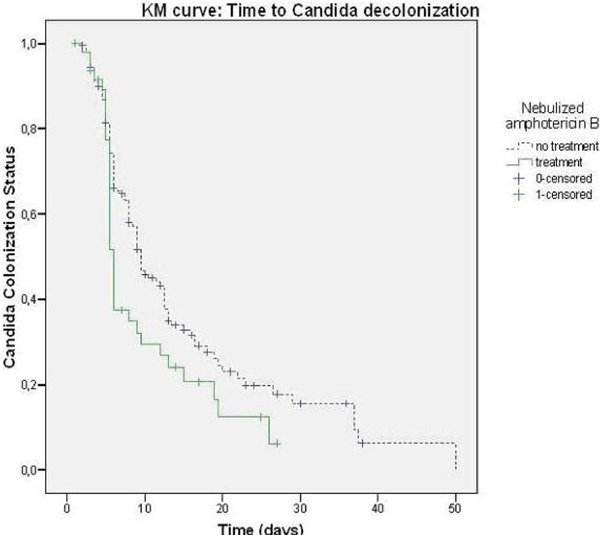


Out of a total of 2,948 patients who were admitted for at least 72 hours, 288 were colonized with *Candida*. Concurrent systemic antifungal treatment during the colonization period was administered to 27 of these patients, who subsequently were excluded, leaving 261 patients for analysis. Decolonization occurred in 36 of 48 (75%) and 112 of 213 (53%) patients who received or did not receive NAB, respectively (HR 1.51; 95% CI = 1.03 to 2.20). Median time to decolonization was 6.0 (IQR 5.6 to 6.4) and 9.0 days (IQR 8.1 to 10.9) in patients receiving and not receiving NAB, respectively (log-rank test, *P *= 0.03) (Figure 1). Adjustment for age, sex and time from ICU admission to acquirement of Candida did not alter the results.

## Conclusion

Inhaled amphotericin B treatment in mechanically ventilated patients with acquired *Candida *colonization of the lower respiratory tract significantly increases the rate of decolonization.

